# Health care workers’ perceptions of point-of-care testing in a low-income country—A qualitative study in Southwestern Uganda

**DOI:** 10.1371/journal.pone.0182005

**Published:** 2017-07-27

**Authors:** Reza Rasti, Deborah Nanjebe, Jonas Karlström, Charles Muchunguzi, Juliet Mwanga-Amumpaire, Jesper Gantelius, Andreas Mårtensson, Lourdes Rivas, Francesc Galban, Philippa Reuterswärd, Helene Andersson Svahn, Helle M. Alvesson, Yap Boum, Tobias Alfvén

**Affiliations:** 1 Department of Public Health Sciences, Karolinska Institutet, Stockholm, Sweden; 2 Sachs’ Children and Youth Hospital, South General Hospital, Stockholm, Sweden; 3 Epicentre Mbarara Research Center, Mbarara, Uganda; 4 UNICEF, Health Section, New York, United States of America; 5 Mbarara University of Science and Technology, Mbarara, Uganda; 6 Division of Proteomics and Nanobiotechnology, KTH Royal Institute of Technology, Science for Life Laboratory, Stockholm, Sweden; 7 Department of Women’s and Children’s Health, International Maternal and Child Health (IMCH), Uppsala University, Uppsala, Sweden; Academic Medical Centre, NETHERLANDS

## Abstract

**Background:**

Point-of-care (POC) tests have become increasingly available and more widely used in recent years. They have been of particular importance to low-income settings, enabling them with clinical capacities that had previously been limited. POC testing programs hold a great potential for significant improvement in low-income health systems. However, as most POC tests are developed in high-income countries, disengagement between developers and end-users inhibit their full potential. This study explores perceptions of POC test end-users in a low-income setting, aiming to support the development of novel POC tests for low-income countries.

**Methods:**

A qualitative study was conducted in Mbarara District, Southwestern Uganda, in October 2014. Fifty health care workers were included in seven focus groups, comprising midwives, laboratory technicians, clinical and medical officers, junior and senior nurses, and medical doctors. Discussions were audio-recorded and transcribed verbatim. Transcripts were coded through a data-driven approach for qualitative content analysis.

**Results:**

Nineteen different POC tests were identified as currently being in use. While participants displayed being widely accustomed to and appreciative of the use of POC tests, they also assessed the use and characteristics of current tests as imperfect. An ideal POC test was characterized as being adapted to local conditions, thoughtfully implemented in the specific health system, and capable of improving the care of patients. Tests for specific medical conditions were requested. Opinions differed with regard to the ideal distribution of POC tests in the local health system.

**Conclusion:**

POC tests are commonly used and greatly appreciated in this study setting. However, there are dissatisfactions with current POC tests and their use. To maximize benefit, stakeholders need to include end-user perspectives in the development and implementation of POC tests. Insights from this study will influence our ongoing efforts to develop POC tests that will be particularly usable in low-income settings.

## Introduction

In recent years, several medical point-of-care (POC) diagnostic tools have been rolled out for programmatic use toward improved diagnosis and disease surveillance in low-income countries [1]. Weak infrastructure, shortage of skilled personnel, and restricted availability of laboratory-based clinical management of patients are major factors behind the lack of qualified health care and the poor outcome of disease in settings with limited resources [2, 3]. POC tools can be defined as tests that are performed near the patient or treatment facility, and have rapid time-to-results, allowing for results to immediately influence patient management [4]. The widespread use of POC tests—such as those for malaria and HIV—in low-income countries could be argued to have risen from previously unmet needs for diagnostic capabilities [5–7]. Some of those needs were predicted to be filled by POC tests early in their large-scale rollout [8]. In endemic areas, POC tests targeting only a few of the highest-burden infectious diseases (malaria, HIV, syphilis, tuberculosis and lower respiratory tract infections) are estimated to have the potential to improve clinical management and thereby save more than one million lives annually [9–11]. Hence, the emergence of POC tests creates the potential for vast improvements in the public health of low-income countries [12]. Further, the emergence of low-cost, easy-to-use rapid POC diagnostic tools has allowed for policies, such as the ‘test-and-treat’ guidelines presented by the World Health Organization (WHO) [13], to be implemented in various regions. Thus, there is potential for POC tests to introduce basic laboratory-based diagnostics to settings without previous capabilities of such [1], as well as to play a role in large national or regional programs tackling various public health issues.

However, as most POC tests are developed in high-income countries, they might not be the optimal tools in settings with weak health care infrastructures [5, 12, 14]. Consequently, there are several reports regarding barriers to the successful use and implementation of POC tests in low- and middle-income health systems [15–17]. Based on these findings, requests have previously been given for the development of POC tools to specifically meet the requirements of low-income health systems [14, 16, 17].

Few studies have explored observations on the use of POC tests directly from the perspective of health personnel in low-income settings, and there are just as few reports by developers of POC tools on whether such perspectives have been considered when developing new ones. The aim of this study was to explore and report these perceptions by engaging health care workers (HCWs) in a rural low-income setting in southwestern Uganda. By bringing insights from the field to the bench, we hope to support our team’s ongoing development of novel POC tests usable in low-income settings, as well as future efforts by other researchers and developers.

## Methods

### Study setting

The Republic of Uganda is an East African low-income country, populated by close to 35 million inhabitants of whom nearly 20 percent live in poverty [18]. This study was conducted in the Mbarara District, Southwestern Uganda. The Mbarara municipality is the fifth largest town in Uganda, with approximately 200,000 inhabitants, located 300 km southwest of Uganda’s capital, Kampala [18]. The larger Mbarara District is mainly a rural, farming district with a few pastoralist communities, housing nearly 500,000 inhabitants from 69 different ethnic groups, the top three being Banyankole, Bakiga and Baganda [19]. Mbarara University of Science and Technology, the Mbarara Regional Referral Hospital and the Epicentre Mbarara Research Center—a research institution with advanced diagnostic facilities affiliated with Médecins Sans Frontières—are all located in the town of Mbarara.

The health system in Uganda is divided into six levels, based on the complexity and speciality of service provision: Health Centres (HC) I to IV, Regional Referral hospitals (V), and National Referral hospitals (VI). The Mbarara Regional Referral Hospital is a reference academic hospital for tertiary care in the region, offering free in- and outpatient specialized care. There are several public and NGO supported Health Centre IV clinics in the district, functioning as “mini hospitals” staffed by nurses, midwives, laboratory technicians, senior medical officers and generalist doctors. These clinics offer in- and outpatient care, and have access to laboratorial diagnostics including microscopy and POC tests for diagnosis of malaria, syphilis, pregnancy and HIV. The NGO supported centres engaged in HIV care are also equipped with more advanced tools for measurement of CD4 count and clinical chemistry parameters. Health Centre III clinics are mostly outpatient clinics run by clinical officers or nurses, and are exclusively equipped with POC tests for diagnostics. Health Centre I and II clinics primarily treat minor ailments and have advisory and referral responsibilities with little or no access to diagnostic tools.

### Study design and participant recruitment

A qualitative approach relying on focus group discussions (FGDs) was chosen to explore health care workers’ experiences with and perceptions of POC testing. Key persons from the district health sector assisted in identifying locally practicing HCWs with current or previous involvement in patient diagnostics and management. In October 2014, a total of 54 HCWs were invited through phone calls, e-mails or face-to-face. All who were contacted provisionally agreed to participate, and 50 were ultimately able to do so. These included junior and senior nurses, medical doctors, midwives, laboratory technicians and clinical officers. The participants practiced medicine at different health care levels, from HC III to level IV, and at the regional referral hospital.

In order to minimize the influence of local hierarchical structures on free speech and to promote professional homogeneity [20, 21], participants were divided into seven focus groups, mainly according to profession. However, at the time of focus group discussions, there were two groups that included individuals of other professions yet from the same health centre levels. This was the case in the focus groups called ‘HC III Clinical Officers’ and ‘HC IV Medical Officers’ (Table 1). Even though these compositions were not as intended, they were accepted due to courtesy to the individuals who had taken time off from their clinical work to participate.

**Table 1:** Composition of the focus groups and characteristics of the participants.

	Focus group						
	Residents and Intern Physicians	Lab Workers	Junior Nurses	Senior Nurses	HC III Clinical Officers	HC IV Medical Officers	Paediatricians
	N						
**Group size**	8	8	8	8	7	6	5
**Age (years)**							
21–30	4	4	8	2	4	3	-
31–40	4	3	-	5	3	3	3
41–50	-	1	-	1	-	-	1
51–60	-	-	-	-	-	-	1
**Gender**							
Male	5	6	1	-	4	3	4
Female	3	2	7	8	3	3	1
**Profession**							
Medical doctor	8	-	-	-	-	4	5
Laboratory technician	-	8	-	-	1	-	-
Nurse	-	-	6	8	1	-	-
Midwife	-	-	2	-	-	1	-
Clinical officer	-	-	-	-	5	1	-

Each focus group contained five to eight participants (Table 1). The FGDs were conducted in English in October 2014 at the Mbarara Regional Referral Hospital.

### Data collection process and analysis

An interview guide was developed, based on discussions by the study team and reviews of similar published studies [15, 22], piloted in a FGD session with ‘Residents and Interns’ group and revised into a final version (Table 2). Before the start of each FGD session, participants were informed about the purpose of the study and the definition of POC tests. All sessions were facilitated by a trained moderator. Two other investigators attended the sessions, providing clarification on technical or clinical aspects of POC tests, when necessary. The sessions were audio-recorded and transcribed verbatim, resulting in eight hours of recordings and 158 pages of transcribed text [20, 23, 24]. The median FGD duration was 58 (range 47 to 117) minutes.

**Table 2:** Final interview guide used during focus group discussions.

**1) Experiences of using current point-of-care technology**
a. What point-of-care tests do you use today?
b. What is your experience with these tests (what aspects do you value?)?
c. What aspects of the tests do you not like?
d. Are there currently available POC tests that you are aware of but do not use? If so, why?
**2) What is most important when you use point-of-care tests?**
a. What are the most important characteristics of a POC test to you?
b. What do you consider the most undesirable characteristics of POC tests?
c. From your experience—are there different concerns at different levels of the health system, regarding POC tests and testing? If yes, what are the most important differences?
**3) What would an ideal point-of-care test include?**
a. What are the most important clinical problems that POC tests help you solve?
b. How could POC testing improve clinical decision making?
c. In order to set up the ideal POC diagnostic tool, what specific tests would you like to include?
**4) Are there any differences in the need of point-of-care tools between the different health care levels in the district?**
a. Are there different needs on different levels of the health system?
b. Regarding the levels, what would be the most important differences in needs?

A data-driven approach for qualitative content analysis [25] was used for identifying specific meaning units to be abstracted into codes. Upon coding of all seven transcripts, an inventory was made of all obtained codes in which they were compared within and across transcripts, followed by the merger of matching codes and the clarification of vague ones. This process was re-examined within the study team, resulting in repeated revisions until a sense of saturation was established concerning the central questions of the interview guide. The final set of codes was repeatedly sorted into sub-categories and categories, and then divided into domains corresponding to the topics of the interview guide [25]. NVivo 11 software for Mac (QSR International 2015) was used for data analysis.

### Ethical considerations

The study was approved by the Institutional Ethical Review Committee of the Mbarara University of Science and Technology (Ref No. 16/08-14) and the Uganda National Council of Science and Technology in Kampala (Ref No. HS 1712). Study participants provided written informed consent and received a refund of their transportation costs and soft drinks during the FGDs. All discussion quotes were anonymized during data analysis and in the resulting manuscript.

## Results

During data analysis, codes and categories were divided into the following four domains: *used and recognized POC tests; perceptions on the utilization of POC tests; requests for an ideal POC test and its utilization*; and *the role of POC testing in the local health system* [S1 Appendix]. Where the first domain reflects participant knowledge of various POC tests, the latter domains reflect on the use of POC tests.

### Used and recognized POC tests

Each FGD started with participants being asked to name POC tests that were used by them, followed by other POC tests they knew of but did not use. Nineteen different POC tests were identified as being in use by at least one participant in each focus group (Table 3). The five types of POC tests named most frequently were those for malaria, HIV, blood glucose, syphilis and pregnancy. Those for malaria and HIV were acknowledged by all focus groups. Furthermore, the group ‘Lab Workers’ reported a wider range of POC tests, whereas ‘Paediatricians’ reported the narrowest. Even though a clarification of what defines a POC test was given prior to discussions, participants were allowed to freely name types of tests. This resulted in the naming of several diagnostic measures that are generally not considered point-of-care, e.g. x-ray and ultrasound.

**Table 3:** Conditions, biomarkers, methods and other measures for diagnosis and monitoring of disease, that the focus groups characterized as point-of-care and in use by themselves.

	Residents and Interns	Lab Workers	Junior Nurses	Senior Nurses	HC III Clinical Officers	HC IV Medical Officers	Paediatricians
*Malaria*	⦁	⦁	⦁	⦁	⦁	⦁	⦁
*HIV*	⦁	⦁	⦁	⦁	⦁	⦁	⦁
*Blood glucose*	⦁			⦁		⦁	⦁
*Urinalysis*	⦁			⦁		⦁	⦁
*Pregnancy*	⦁	⦁	⦁	⦁	⦁	⦁	
*Syphilis*		⦁	⦁	⦁	⦁	⦁	
*Ultra sound*			⦁				
*Blood slide*				⦁			
*Microscopy of cerebrospinal fluid*				⦁			
*CD4 count*		⦁					
*Meningitis*		⦁					
*Prostate Specific Antigen*		⦁					
*Tuberculosis*		⦁					
*Hepatitis B*		⦁			⦁	⦁	
*Hepatitis C*		⦁					
*Enteric fever*					⦁		
*Brucellosis*						⦁	
*X-ray*			⦁				
*Procalcitonin*		⦁					

At least one mention in groups marked by ⦁

Furthermore, participants were asked to identify other known POC tests that were currently not being used by them (Table 4).

**Table 4:** Other POC tests recognized by focus groups but not used by them.

	Residents and Interns	Lab Workers	Junior Nurses	Senior Nurses	HC III Clinical Officers	HC IV Medical Officers	Paediatricians
*Typhoid*	⦁			⦁			⦁
*Syphilis*	⦁	⦁					⦁
*Rota virus*							⦁
*Herpes simplex virus*							⦁
*Streptococcus group A*							⦁
*Helicobacter pylori*	⦁			⦁	⦁	⦁	
*Brucellosis*	⦁					⦁	
*Transcutaneous bilirubinometer*	⦁						
*Blood gas*	⦁	⦁					
*Human papilloma virus*	⦁						
*Hepatitis B*				⦁			
*Full blood count*				⦁			
*Procalcitonin*		⦁					
*Chlamydia*		⦁					
*Gonorrhoea*		⦁					
*Electrolytes*		⦁					
*Tuberculosis*		⦁			⦁	⦁	
*Enteric fever*					⦁		
*Toxoplasmosis*		⦁					

At least one mention in groups marked by ⦁

The explanations that were given for not using these additional POC tests were due to their expense, issues regarding supply, and that participants perceived themselves non-influential on decisions regarding the availability of POC tests. As exemplified by one paediatrician:

*I think … we are in a government facility so we are in a situation where we use what is given to us and sometimes not in position to ask for many things*.

(Participant 1, focus group Paediatricians)

### Perceptions on the utilization of POC tests

Following the interview guide, participants were asked for their opinions regarding the use of POC tests. These opinions were classified into two categories during analysis: *POC testing strengthens the health system*, by being (1) practical in the local context, and (2) improving patient management; and *current tests do not meet the needs of the health system* due to (a) distrust in test results, (b) improper use of POC tests, and (c) imperfection of POC tests in the local context.

#### POC tests are practical in the local context

There were several aspects of POC tests that participants perceived suitable for local conditions. POC tests were described as easy-to-learn and easy-to-use, allowing others than only trained laboratory technicians to perform testing and interpret results:

*… they are not complicated, they don’t need too much technical knowhow, even if you have never used it before, someone can just show you and you can just use*.

(Participant 3, focus group Senior Nurses)

Also, participants described cases in which the user-friendliness of POC tests enabled a relocation of disease monitoring from health facilities to the homes of patients, hence reducing the workload at health facilities. As one medical doctor explains:

*… let’s say they have diabetes, you give them a glucometer and you tell them that when they eat food, they have to monitor their blood sugar. If it is this high, rush to the hospital. So it eases the work of a medical worker*.

(Participant 2, focus group Residents and Interns)

There were several characteristics of POC tests that the focus group participants recognized as especially practical for the local health care, e.g. requiring fewer resources (being affordable and maintenance-free), not needing skilled personnel to operate, and allowing diagnostics even without having access to traditional laboratories:

*They* [POC tests] *are very helpful because where the facilities are not enough they really help, like you may not be having a microscope and you use the test and it helps you to manage the patient*.

(Participant 3, focus group Residents and Interns)

#### POC tests improve management of patients

Besides considering POC tests as practical, participants perceived their accuracy and their rapid time-to-results as essential for improved patient management. Almost all focus groups credited POC tests with simpler and faster differential diagnosis, enabling more adequate clinical decision-making, improved treatment targeting, and lessened burden of drug resistance. As described by a medical doctor:

*I think they help a great deal in diagnosis and narrowing our definition of differential diagnosis. For example, if a patient comes in and you do an HIV rapid test diagnostic and it’s positive, then you can easily compare it with the clinical picture and we come out with a different range of differential diagnosis*.

(Participant 7, focus group Residents and Interns)

The POC tests for blood glucose and malaria were highlighted as being especially useful when managing medical emergencies. As one senior nurse illustrates, POC tests can have life-saving impact:

*If a patient is in coma, and probably it’s due to hypoglycaemia, you will use a glucose strip test, and you check their glucose levels. And you will be able to give a bolus of like glucose and you will be able to resuscitate that patient that way. So otherwise, you wouldn’t know the cause and probably you would miss that out*.

(Participant 3, focus group Senior Nurses)

#### Distrust in test results

Despite the previously described perceived accuracy of POC tests, all groups expressed doubts regarding their trustworthiness. Some of these doubts concerned the diagnostic accuracy of tests, and others concerned the robustness of POC test assays, or the clarity of their results (Table 5).

**Table 5:** Codes with corresponding quotes related to the category ‘Distrust in test results’.

Test devices are overly sensitive to handling and storage	*Sometimes they say if it has been in your bag*, *it has been exposed to heat*, *you use it and you find it will not show anything* (Participant 3, focus group Junior Nurses)
POC tests have low sensitivity and specificity	*… you can test for malaria*, *the RDT* [rapid diagnostic test] *is negative*, *the clinical symptoms are malaria*, *and you do a BS* [blood smear microscopy] *and they find it positive* (Participant 6, focus group Senior Nurses)
Some tests falsely signal previous infections as active ones	*… the challenge I have with these point-of-care tests is that some of them remain positive even after the infection has been cleared*. (Participant 3, focus group Lab Workers)
Test results can be unclear, allowing disagreement between users	*One technician will say it is faint and in most cases when it is faint*, *people will begin to argue about it*. *One will say negative and another one will say positive*. (Participant 8, focus group Lab Workers)

Furthermore, the participants expressed suspicions of counterfeit test devices being delivered to their health facilities, adding to their distrust.

#### Improper use of POC tests

Despite previous assessments that POC tests were easy to use, participants widely criticized what was perceived as knowledge gaps in the use of POC tests and expressed concerns about their incorrect use, e.g. the use of wrong buffer solutions, or none at all, even when required. This was illustrated by a paediatrician:

…*my personal experience, oftentimes tests are done without buffering and I don’t know what that means… Because I have seen where someone is doing a test and is not bothered with the buffer*.

(Participant 2, focus group Paediatricians)

The described benefit of the simplicity of POC testing, was in some cases also perceived as worrisome, especially in combination with a perceived overreliance on POC test results. This was described by participants as causing erroneous decision-making based on false results and leading more accurate, but more complicated test methods to be underutilized and neglected.

#### Imperfection of POC tests in the local context

Considering usability in the local context, there were several aspects of POC tests that the participants deemed imperfect. Some of these were related to the accessibility of POC tests; others to health finance decisions. Test kits and their required consumables (such as test strips and reagents) were witnessed as often being out of stock, or as expensive and bulky.

Other imperfections were expressed in terms of the limitations of currently used POC tests, such as each test typically being able to analyze only one parameter or pathogen:

*… it will only show you it is malaria and any other infections it doesn’t show you*.

(Participant 3, focus group Junior Nurses)

### Requests for an ideal POC test and its utilization

When the focus groups were asked to think visionary with regard to a future POC test, their suggestions could be divided into three categories of requests for an ideal POC test: *a test adapted to the local context*; *a test with a pre-defined implementation in the health system*; and *a test with the ability to improve the care of patients*. These categories were not internally ranked.

#### A test adapted to the local context

Participants identified several requirements for adapting POC tests to local conditions, including their being resource-efficient and functional in the local environment. Test devices—and their accessories, which, preferably, should not be needed at all or at least be interchangeable between different types of devices—should be low-cost, be capable of providing quick results, and be easy to learn and operate for clinicians as well as some patient groups. Furthermore, participants requested that POC test kits be made available to them at all times. In addition, due to the warm climate and unreliable power supply of the area, participants requested heat stable tests that do not rely on electricity to function.

#### A test with a pre-defined implementation in the health system

Apart from the requested characteristics of ideal POC tests, participants stressed the need for decision makers to clearly define what role tests are to play in the health system and distribute them according to the patient load and relevant epidemiology of the communities in which they are to be used:

*For example, here is American trypanosomiasis. I don’t expect a rapid test for it since we don’t have it. So they should be wired* [delivered] *depending on the diseases in a given community*.

(Participant 7, focus group Residents and Interns)

Participants stressed that intended users ought to be properly trained before large-scale deployment of POC tests. Similarly, addressing previously described distrust in test results, there were demands for clear management protocols for how to proceed from the results. Requests were made for the confirmation of POC test results by more conventional and reliable diagnostic methods. The following example by a paediatrician urges this request to avoid inaccurate—and potentially life-altering—diagnoses and treatments:

*And then they also have to understand the limitations of the test. We have seen that with HIV also. There is an algorithm. So the first test* [POC test] *… can give you false positives. The false positives then have to be confirmed by the second test. …But many health units were doing the first test only and giving the result. Telling people that they are positive, while some of them were not positive. And this is a life-changing diagnosis. But they were not aware of it and most times those people went to a health unit where ART* [antiretroviral therapy] *was available and they got on to ARTs without confirming. Certainly they turned out to be negative*.

(Participant 4, focus group Paediatricians)

#### A test with the ability to improve the care of patients

The importance of POC tests to the improved care of patients was highlighted by all groups. It was underlined that POC tests should have high diagnostic accuracy, and provide trustworthy and indisputable results. Further, the ideal POC test should ultimately support management of both communicable and non-communicable diseases, and require minimal or even non-invasive techniques for sample collection:

*If it is a child who has maybe a suspicion of meningitis, it should be using methods that are non-invasive and comfortable for our patients*.

(Participant 6, focus group Lab Workers)

With regards to communicable diseases, the groups requested POC tests capable of distinguishing between causes of the infections, thus allowing for targeted treatment.

Furthermore, the groups requested an ideal POC test to have the capacity to measure certain biomarkers of clinical interest (e.g. full blood profile, organ function and inflammatory biomarkers) as well as the ability to diagnose specific infectious diseases (Table 6).

**Table 6:** Specific requests for what ideal POC tests should be able to measure, state or diagnose.

	Residents and Interns	Lab Workers	Junior Nurses	Senior Nurses	HC III Clinical Officers	HC IV Medical Officers	Paediatricians
*Distinguish between bacterial/non bacterial infection*	⦁	⦁		⦁		⦁	⦁
*Microbiology of most common infections*	⦁	⦁				⦁	⦁
*Respiratory tract infections*		⦁				⦁	⦁
*Etiology of fever*		⦁					
*CNS infections*		⦁		⦁			
*Etiology of diarrhea*	⦁	⦁				⦁	
*Brucellosis*		⦁	⦁		⦁		
*Ebola*				⦁	⦁	⦁	
*Enteric fever*					⦁		
*Fungal infections*		⦁					⦁
*Hepatitis B*			⦁		⦁		
*HIV*	⦁		⦁	⦁			
*Human Papilloma Virus*	⦁						
*Malaria*	⦁	⦁	⦁	⦁		⦁	
*Marburg virus*				⦁	⦁	⦁	
*Syphilis*		⦁		⦁			
*Tuberculosis*		⦁	⦁		⦁	⦁	
*Throat infection*							⦁
*Toxoplasmosis*		⦁					
*Typhoid*		⦁	⦁				
*Severity of infection*		⦁	⦁			⦁	
*Drug resistance*		⦁					
*Basic organ functions*	⦁						⦁
*Blood gas*	⦁						⦁
*Blood group*				⦁			
*Blood slide*							⦁
*Blood glucose*	⦁			⦁			⦁
*C-reactive Protein*	⦁						
*Electrolytes*							⦁
*“Everything”*							⦁
*Full blood count*							⦁
*Haemoglobin*	⦁	⦁		⦁			
*Immunodeficiency*							⦁
*Procalcitonin*	⦁						
*Sickle cell anemia*	⦁			⦁			

At least one mention in groups marked by ⦁

### The role of POC testing in the local health system

A topic that caused vibrant discussions within the groups was at what level of care the need for POC tests is highest, and their potential role within the health system. Some participants proposed a higher distribution to secondary and tertiary care facilities, arguing that these went to managing larger numbers of patients and a broader span of illnesses.

Others stressed a greater importance for POC tests at primary care, due to the lack of alternative diagnostic methods at these facilities. Furthermore, participants in several of the focus groups from both lower and higher levels suggested that the role of POC tests at lower levels should primarily be to aid in determining the severity of disease and assist decisions regarding referral. At these lower levels, uncomplicated test devices for the most common illnesses were described as needed.

The groups considered all levels to be in need of POC tests for infectious diseases. However, they indicated that the need varies for other tests; different clinical specialties have different needs for specific POC tests, and the different levels in the health system have different capacities for managing test results.

Although it was not within the immediate scope of this study, participants expressed concerns, or rather frustrations, about the organization of the local health system, e.g. patient flows not following intended pathways through the ladder of referral. This perceived problem stirred in some groups the need for POC tests for a broad range of conditions, even at the higher levels of care. As exemplified by a paediatrician:

*Sometimes, in the health system of Uganda has given people the mandate to walk in anywhere with whatever disease they have because much as people have bigger diseases and may come to us, the hospital may fail on those bigger diseases and say go back to the lower centres. So here you are with a child who is going to get unconscious and the one who has flu and they will all walk through the same* [entrance]. *Because there is no sieve anywhere*.

(Participant 1, focus group Paediatricians)

## Discussion

This is, to our knowledge, the first qualitative study assessing perceptions of the use of POC tests among HCWs in low-income countries, after the large rollout programs of recent years. Our findings show that the participating HCWs are accustomed to using POC tests in their everyday clinical work. They witness tests being used for clinical decision-making by all included professions, which highlights the importance of POC tests to the local health system.

The focus group participants identified a range of different POC tests in use, the top five being those for malaria, HIV, syphilis, blood glucose and pregnancy, in line with surveys in other parts of Uganda [26]. Participants also showed knowledge of an even wider range of POC tests that were marketed, even though they did not use them. The main reasons that were given for not using these tests were their cost and that participants perceived themselves as being non-influential in decisions regarding test availability.

Furthermore, this study shows an agreement among participants that POC tests strengthen their health system and improve the health care provided in their district. Aspects of POC tests that were especially valued were their user-friendliness, their rapid time-to-results, their ability to conserve resources and their ability to improve clinical decision-making regarding referral or targeted treatment. At the higher levels of care, POC tests were also perceived as being helpful in differential diagnostics and the management of medical emergencies. However, POC tests were especially noted as enabling lower levels of the health system with diagnostic capacities that would otherwise be lacking, something also appreciated in other low-income settings [27].

Even though the participants perceived POC testing as being critical for improved patient management, they assessed the POC tests in their current forms as imperfect and as not fulfilling the needs of the local context. This was made evident through the participants expressing distrust in the accuracy of test results, witnessing testing procedures conducted wrongly, describing the lack of training and knowledge among users of POC tests, assessing test kits as being expensive and bulky, and finding them frequently unavailable when needed. The contradictory expressions of the participants regarding trustworthiness of POC tests, indicate differences in perceived accuracy between the various available test kits, as well as a lack of confidence in their proper use. Where tests for e.g. blood glucose were described as easy and trustworthy enough to be used in emergencies, or even by patients themselves, other tests—such as the ones for infectious diseases—were described as complicated to perform, and their results often inaccurate or unclear.

Furthermore, participants found limitations of POC tests in that they were single-analytic and that more reliable diagnostic methods were being neglected in favor of easy-to-use POC tests. Some of these perceived downfalls of POC tests and their use have previously been described in the same region [28]. Other inconveniences identified by this study have been described elsewhere, including difficulties in ensuring proper logistical processes, old infections being falsely signaled as active, inadequate training of end-users and insufficient knowledge of the use of POC tests, dubious quality of deployed tests, and test kits being unavailable when needed [27, 29–32]. In some reports, these unappreciated aspects of POC testing have been described as barriers inhibiting successful implementation of POC tests into health systems of low- and middle-income countries [16, 33], as well as in high-income countries [34]. The latter conceivably signal issues regarding design and manufacturing, that ought to be addressed by developers, as well as inadequate policies regarding the use of POC tests being common in low-, middle- and high-income countries.

Complicated testing procedures; misconceptions or knowledge gaps of POC tests; incorrect handling; incoherent interpretation of test results; and clinical assumptions based on false or incorrectly interpreted test results, were some of the adverse aspects of POC tests and their use, described in this study conducted in 2014. The same aspects were described already in 2007, as early Ugandan experiences from the—at the time—newly rolled-out POC tests for malaria [35].

When asked to be visionary regarding the *ideal* POC tool, participants called for tests and strategies for their use that retained all the beneficial characteristics of current POC tests, while at the same time adjusting for the ‘imperfect’ properties of POC tools, as described above. Requests given for specific POC tests and test characteristics (low-cost, quick, easy-to-learn, high availability of tests, tests not needing extra consumables/accessories) are consistent with the ‘ASSURED’ criteria by the WHO [36], as well as with recommendations by others regarding the ideal characteristics of POC tools for low-income settings [14, 36]. Besides these specific requests, the focus groups stressed the need for pre-definition of the use of POC tests and made general requests for tests that improve the care of their patients.

Many participants demanded clear action plans, or clinical protocols, for the use of POC tests in patient management. These requests were not restricted to the sentiments of our participants, as they have been recognized by others and have been included in recommendations for POC test developers, policy makers, health providers and funders when seeking to integrate POC tests in health systems [16, 33, 37]. Other authors have suggested integrating the use of POC tests into existing protocols for patient management—such as the Integrated Management of Childhood Illness—in order to avoid some of the incorrect testing procedures described above [28].

To ensure improved care of patients, participants highlighted that POC tests ought to give accurate and trustworthy results. They called for tests for communicable and non-communicable conditions, and emphasized the need for POC tests that can distinguish between etiological causes of infection, hence targeting treatments. Requests were also made for testing procedures with less discomfort for patients, ideally completely non-invasive procedures.

The focus groups showed a disagreement regarding at what level of the local health system that POC tests could be optimally used. What could be derived from discussions was that there is a high demand for POC diagnostics—and especially for infectious diseases—at all levels of the health system. Higher levels, despite their high access to advanced infrastructure and skilled human resources, still need POC tests, as they manage patients with a wide range of conditions, from advanced diseases that cannot be managed elsewhere to primary care cases that end up at the hospital due to what is perceived as a failure of the referral systems. This frustration is not unique to the low-income context of this study but is also shared by health providers in high-income countries [38–41].

Lower levels are dependent on POC tests, and most benefited by them, as their access to alternative diagnostic methods is often limited or even non-existent. Here POC tests are described as instrumental for determining the severity of disease and making decisions regarding referral or treatment of the most common conditions.

It is noteworthy that this study recognizes key difficulties in the proper use of POC tests by HCWs that were previously identified in the early stages of test deployment in Uganda [35]. This could be an indication of insufficient evaluation of the use of POC tests and inadequate training in their proper use since being introduced to the Ugandan health system. However, it could also indicate the difficulty in developing, deploying, supporting and implementing a POC test that can be deemed as ‘perfect’ in all aspects.

The suboptimal performance of many POC tools, as well as the imperfect implementations of their use, impedes their true potential. Lack of regulatory policies regarding quality of diagnostic POC tests marketed in low-income countries has led to the spread of substandard POC tests [5], something also echoed in this study. Successful implementation of POC tools into the health systems of low-income settings are highly dependent on policies regarding their use, and recommendations on how to achieve this have been given by others [14, 42, 43]. Recommendations from this study are that policy and decision makers ought to define the purpose and role of POC tools in advance; evaluate their use and field performance after introduction; ensure proper training of intended end-users; and draw clear action plans—perhaps integrated into existing protocols—for the use of POC tests and management of test results. Equally important is that developers and manufacturers of diagnostic POC tools, in order to ensure high usability, ought to take into account the context in which their products are to be used.

Even though many developers of POC tests for low-income settings are guided by various general criteria—such as ASSURED—we recommend that they seek direct insight into the actual circumstances of the intended context, which is a recommendation supported by other authors [16]. Tentatively, this should be done by making the effort to gather opinions and requests of end-users through studies similar to ours, or by seeking firsthand experience through field visits, as reported by Garcia et al [44], or preferably through a combination of both approaches. Meanwhile, the findings of this study adds new knowledge on the use of and view on POC tests among HCWs in Uganda. Furthermore, the gathered requests for ideal POC tests from our study, confirm and add on to findings of other reports, and have allowed our team of investigators to pursue an informed development of novel POC tools for microbiology of infectious diseases, especially amenable to low-income settings.

### Limitations of the study

The main limitation of this study is that the focus groups are not representative of health care workers of *all* low-income settings. There are substantial cultural, political, environmental and epidemiological differences between a low-resource setting in sub-Saharan Africa as compared to, for example, Central Asia. Also, it is common to have varieties within countries, if not of the sorts noted above, then these often occur due to socioeconomic differences. Furthermore, the generalizability of this study to the district where it has been conducted is limited by its non-inclusion of participants from local privately funded health facilities, where POC tests are also being used. However, several of the findings of this study are shared with similar studies in higher-income settings countries, indicating that they might be transferrable to other settings of similar access to POC tests, irrespective of socioeconomic context. Even though it was intended to group participants strictly according to profession, this was not the case in two out of seven focus groups. Despite this, we believe that all participants could speak their mind in an open fashion. Furthermore, the choice not to include other data collection methods than focus group discussions (e.g. personal in-depth interviewing) in this study, inhibits data triangulation. Also, by only using qualitative methods for data collection, and not including investigative data (e.g. seeking information directly from the health centres regarding their access to POC tests) allows for biased data, as the data is based on the recollections of participants and not necessarily on factual circumstances. This is exemplified by no group identifying rapid tests for Haemoglobin (Tables 3 & 4), even though it is available in the district. However, we believe that the main aim of this study—exploring *perceptions* of the *use* of POC tests—has been well attained by the reliance on the used qualitative methods.

## Conclusion

Point-of-care tests are widely used for clinical management of a range of medical conditions in southwestern Uganda, where they are appreciated for strengthening the health system.

The study identifies specific requests for *ideal* POC tests and proposes a scenario in which their characteristics are adapted to local circumstances, their use is protocol based, and their role and place in the health system is pre-defined. However, end-users show dissatisfaction with the use and performance of current POC tests, deeming them imperfect. Issues of distrust in test accuracy, limitations in their characteristics, and testimonies regarding a lack of training on the POC tests and their incorrect use inhibit their full potential.

Local health care workers call for less invasive and more accurate POC tests that cater to the differing needs of all levels of their health system. For these requests to be fulfilled, there is a need for test developers and policy makers alike to seek insight into the conditions and requests of intended end-users in low-income countries, to properly train and prepare health providers, and to regularly re-evaluate the use of introduced of POC tests.

## Supporting information

S1 Appendix Code structure point-of-care Uganda Rasti et al. An overview of the coding structure during data analysis.
(XLSX)
